# Infection Kinetics and Phylogenetic Analysis of vB_EcoD_SU57, a Virulent T1-Like *Drexlerviridae* Coliphage

**DOI:** 10.3389/fmicb.2020.565556

**Published:** 2020-11-16

**Authors:** Shazeeda Koonjan, Fredrik Seijsing, Callum J. Cooper, Anders S. Nilsson

**Affiliations:** ^1^Department of Molecular Biosciences, The Wenner-Gren Institute, Stockholm University, Stockholm, Sweden; ^2^School of Pharmacy, Pharmaceutical and Cosmetic Sciences, Faculty of Health Sciences and Wellbeing, University of Sunderland, Sunderland, United Kingdom

**Keywords:** T1-like bacteriophage, phage virulence, phage infection kinetics, phage phylogenetics, *Drexlerviridae*

## Abstract

The morphology, infection kinetics, genome sequence and phylogenetic characterization of the previously isolated bacteriophage vB_EcoD_SU57 are presented. The phage vB_EcoD_SU57 was isolated on *Escherichia coli* strain ECOR57 from the *E. coli* reference collection and was shown to produce four mm clear plaques with halos. Infection kinetics, as assessed by one-step growth analyses, suggest that vB_EcoD_SU57 is a virulent phage with an adsorption rate of 8.5 × 10^–10^ mL × min^–1^, a latency period of 14 min, and a burst size of 13 PFU per bacterium. Transmission electron microscopy confirmed vB_EcoD_SU57 to be a phage that used to be classified as a *Siphoviridae* phage. Bioinformatics analyses showed that the genome was 46,150 base pairs long, contained 29 genes with predicted protein functions, and 51 open reading frames encoding proteins with unknown function, many of which were gathered in clusters. A putative tRNA gene was also identified. Phylogenetic analyses showed that vB_EcoD_SU57 is a *Braunvirinae* phage of the newly formed *Drexlerviridae* family and closely related to T1-like *E. coli* phages vB_EcoS_ACG-M12 (Guelphvirus) and Rtp (Rtpvirus) as well as the unclassified phages vB_EcoS_CEB_EC3a and ECH1.

## Introduction

T1-like phages have an icosahedral head of about 60 nm and a slender tail of about 150 nm, placing them in the newly formed family of tailed phages, *Drexlerviridae*, which consists of T1-like phages from the *Siphoviridae* family (German et al., 2006; Adriaenssens et al., 2020). Phage T1 itself was one of the first phages to be studied in detail as it was one of the original “type” phages selected by Drexler (1988). Phage T1 was shown to have unique properties and soon became infamous for its ability to invade microbiological laboratories, ruining many experiments by lysing the standard *Escherichia coli* strain K12. This is the reason why most laboratories today use T1 resistant *E. coli* strains. To date, many more relatives to T1 have been isolated and their genome sequenced, and it appears as if close relatives to phage T1 are only to be found to infect *E. coli* and *Shigella* species. Phages of this coherent group are classified into the genus *Tunavirus*, and together with other related T1-like phages classified into two other genera into the subfamily *Tunavirinae*. The *Drexlerviridae* family consists of three additional subfamilies, holding many genera of T1-like phages, all of which were isolated utilizing gammaproteobacteria as hosts (Niu et al., 2014; Adriaenssens et al., 2020).

T1-like phages are virulent and have high adsorption rates, and as such have gained attention as candidate phages in the development of phage therapy (Kutter et al., 2010; Abedon et al., 2011; Divya Ganeshan and Hosseinidoust, 2019). The adsorption rate of phage T1 is around 3 × 10^–9^ mL × min^–1^, which is considered to be among the highest rates of phage adsorption, and the time from infection to lysis of the bacterium is short, 15–20 min (Drexler, 1988; German et al., 2006). These properties would prove advantageous for phage therapy as they theoretically lead to a fast elimination of an infecting bacteria, provided that the phage dose reaching the bacteria is high enough.

Phage vB_EcoD_SU57 (henceforth referred to as SU57) was isolated from a sewage water treatment works outside of Stockholm, Sweden (Khan Mirzaei and Nilsson, 2015). It should be noted that SU57 was previously mislabeled as vB_EcoP_SU57. Its primary host of isolation was the ECOR57 strain from the *E. coli* reference collection (ECOR) (Ochman and Selander, 1984). Earlier studies of this phage showed that it had a narrow host range effectively infecting only its strain of isolation, as well as an *E. coli* ESBL and a *Salmonella* strain from the SARB collection with comparable efficiency (efficiency of plating, EOP > 0.5), and two other ECOR strains with a considerably lower EOP (Boyd et al., 1993; Khan Mirzaei and Nilsson, 2015). SU57 was also shown to be a virulent phage capable of lysing its host strain in 17 min and to form clear plaques on a lawn of host bacteria (Khan Mirzaei and Nilsson, 2015). In this study, SU57 has been characterized based on its morphology, infection biology kinetics, genomic composition, and phylogenetic relatedness to other phages.

## Materials and Methods

### Bacterial Strains and Growth Conditions

The *E. coli* ECOR standard reference collection (Ochman and Selander, 1984) was kindly provided by Diarmaid Hughes and Dan Andersson of Uppsala University, Sweden. Bacterial cultures were routinely grown in Miller’s lysogeny broth (LB; Neogen, United States) with shaking at 150 RPM or on tryptone yeast agar (TYA; Biolife Italiana, Italy) on plates incubated at 37°C.

### Phage Propagation and Purification

Phage SU57 was obtained from stocks based at Stockholm University and routinely propagated on ECOR57. SU57 was previously isolated from Käppala waste water treatment plant located 15 km East of Stockholm, Sweden, in November 2010 (Khan Mirzaei and Nilsson, 2015). For phage enrichments, fresh media was inoculated with 100 μL overnight bacterial cultures and allowed to grow to mid-log phase at 37°C with shaking or until optical density at 600 nm (OD_600_) was 0.6. SU57 was enumerated by using the agar overlay (OA) method with 65% w/v (22.75 g/L) TYA as previously described (Gratia, 1936; Kropinski et al., 2009). Concentrated stocks of SU57 were produced using polyethylene glycol (PEG) precipitation, modified from Sambrook and Russell (2006). In brief, crude phage lysate suspensions were centrifuged at 3864 × *g* for 10 min and the supernatant passed through a sterile 0.45 μm syringe filter (Sarstedt Filtropur, Germany). Phages were precipitated by adding solid NaCl and PEG8000 (Acros Organics, Germany) to the partially purified suspension to final concentrations of 1M and 10% (w/v), respectively, and then stored at 4°C for 2 weeks. Following refrigeration, phages were centrifuged at 11,000 × *g* for 1 h at 4°C and the pellet re-suspended in 50 mL phosphate buffered saline (PBS). The phage content was enumerated using the OA method as previously described (Kropinski et al., 2009).

### Plaque Morphology Determination

SU57 plaque morphology was determined using ECOR57 as the host bacterium and the OA method (Kropinski et al., 2009). In brief, SU57 phage stock was serially diluted 1:10 in PBS. Three mL of OA was inoculated with 100 μL of overnight host bacteria culture (approximately 10^8^ CFU mL^–1^) and 100 μL of 10^–7^ serially diluted SU57. The OA was poured over the surface of a pre-prepared TYA plates and incubated for approximately 18 h at 37°C. Plaques were imaged using a Samsung SM-G930T camera. Eighty plaque diameters were calculated using the ImageJ software (Schneider et al., 2012).

### Transmission Electron Microscopy (TEM)

Polyethylene glycol purified SU57 (2.5 × 10^9^ PFU mL^–1^) was negatively stained with 1% (w/v) uranyl acetate (Broers et al., 1975) and visualized on a TECNAI G2 Spirit Bio TWIN, 80 kV (FEI Company). Dimensions of four SU57 virions were measured at 30,000 × magnification and analyzed with ImageJ (Schneider et al., 2012).

### One-Step Growth Curve Analysis

The adsorption rate constant [the likelihood of a single phage infecting and adsorbing to a single bacterium over time (Abedon, 2012a)], latency period [the time it takes a phage to reproduce inside an infected host cell (Sinha et al., 2018)], and burst size [the ratio of the phage titer after lysis to phage adsorbed titer (Hyman and Abedon, 2009)] of SU57 were determined using the one-step growth curve protocol adapted from Hyman and Abedon (2009). In brief, 50 mL of LB was inoculated with 50 μL of ECOR57 and incubated at 37°C with shaking until the bacteria reached mid-log phase (OD_600_ 0.6). Once OD_600_ 0.6 was obtained, bacterial suspension was removed so the final volume of the bacterial suspension used for the experiment was 44 mL. One mL of SU57 phage stock (2.5 × 10^9^ PFU mL^–1^) was added to mid-log phase bacteria (approximately 4 × 10^8^ CFU mL^–1^) at a multiplicity of infection (MOI) of 0.14 and mixed by swirling (*T* = 0). One mL aliquots were withdrawn at 2 min intervals for the first 30 min and 5 min intervals for the remaining 30 min and their OD_600_ was determined with the same spectrophotometer used to determine the bacterial inoculum OD_600_. An additional 500 μL aliquot was removed at each time point and centrifuged at 6000 × *g* for 1 min to pellet the bacteria. Using the supernatant, 1:10 serial dilutions in PBS was done to determine the phage content during each time point. All experiments were performed in triplicates. The adsorption rate constant was determined using the formula shown below (Hyman and Abedon, 2009) where *N* is the bacterial density, *P*_o_ and *P* are the starting and ending phage titers, *k* is the adsorption rate constant, and *t* is the time in minutes over which adsorption occurs:

k=-ln⁢(P/Po)/Nt

It should be noted that the adsorption rate constant was determined using only two time points (*T* = 0 and *T* = 4) and not calculated from the curve entirety. The burst size was calculated by dividing the phage titer after the first burst (approximately at 26 min) with the number of adsorbed phages (initial phage concentration at *T* = 0 minus phage concentration at *T* = 4).

### Phage DNA Extraction

SU57 DNA was extracted from suspensions containing a minimum of 1 × 10^8^ PFU mL^–1^ using Norgen Biotek phage DNA isolation kit (Nordic BioSite AB, Sweden) with an additional DNAse I treatment according to the manufacturer’s instructions. Prior to sequencing, DNA concentration was quantified by fluorometry on a Qubit 2.0 (Invitrogen, Thermo Fisher, Sweden) and purity assessed by gel electrophoresis.

### Genome Sequencing and Bioinformatics of the SU57 Genome

The genome of SU57 was sequenced using Pacific Biosciences Sequel II Single Molecule Real-Time sequencing at SciLifeLab, NGI, Uppsala, Sweden. Reads were assembled using Canu 1.8 with default settings and SPAdes 3.9.0 on the Galaxy@Pasteur platform^1^ (Bankevich et al., 2012; Koren et al., 2017; Mareuil et al., 2017; Afgan et al., 2018). Open reading frames (ORFs) and genes were predicted and annotated using Glimmer3 prediction in the Geneious 6.1.8 software package (Geneious^2^; Delcher et al., 2007). Inferred amino acid sequences were compared against the National Center of Biotechnology Information (NCBI) non-redundant (nr) protein sequences database specific to *Caudovirales* with the Basic Local Alignment Search Tool (BLAST) BLASTx software (Altschul et al., 1997). tRNA genes were detected using ARAGORN 1.2.38 (Laslett and Canback, 2004). An *E. coli* type of origin of replication (oriC) sequence was sought for by searching for a similar sequence in the SU57 nucleotide sequence. Hypothetical bacterial σ^70^ promoter regions and rho-independent terminators were found using BPROM (Softberry, Mount Kisco, NY, United States) (BPROM; Solovyev and Salamov, 2011) and the ARNold Web server (ARNold^3^; Naville et al., 2011), respectively. Promoter regions were identified based on the spacing between −10 and −35 elements being 17 nucleotides and the *E. coli* consensus sequences: −10: TATAAT and −35: TTGACA (Estrem et al., 1999; Guzina and Djordjevic, 2015). Ribosomal binding sites (RBS) were identified based on the Shine–Dalgarno sequence AGGAGG in untranslated regions (UTR) approximately 5–9 nucleotides upstream of an identified ORFs start codon (Shine and Dalgarno, 1974; Ma et al., 2002). Genomic guanine-cytosine (GC) content and restriction enzyme sites were found using the European Molecular Biology Open Software Suite (EMBOSS) geecee and restrict programs, respectively (EMBOSS: geecee^4^; EMBOSS: restrict^5^; Rice et al., 2000). PhageTerm was used to determine the genome termini and packaging mechanism (Garneau et al., 2017).

### Restriction Digest Analyses

In order to determine the circular permuted nature of SU57 genome, restriction digest analyses were performed. Approximately 300 ng of SU57 DNA was digested with 1 μl of Fast Digest *Sac*I, *Apa*I or *Xho*I, and double digests using Fast Digest *Sac*I and *Apa*I according to a modified manufacturer’s protocol (Thermo Scientific; Vilnius, Lithuania). In brief, samples were incubated in a 37°C water bath for 17.5 min followed by a 10 min enzyme inactivation at 80°C. Samples were analyzed with gel electrophoresis using a 0.7% (w/v) SeaKem^®^ LE agarose (Lonza; Rockland, ME, United States) gel with 0.1X of GelRed^®^ Nucleic acid stain (10,000X in DMSO; EMD Millipore Corp., United States) and 1X Tris-acetate-EDTA running buffer. The system was run for 150 min at 70 V and the gel was imaged using Bio-Rad Laboratory’s Image Lab software version 5.0. Samples were additionally analyzed using pulse field gel electrophoresis (PFGE). Using a modified procedure from Paul et al. (2005), PFGE was performed using a 1% PFGE agarose gel (Sigma-Aldrich; Steinheim, Germany) and 1X Tris-borate-EDTA. A CHEF-DR II PFGE system (Bio-Rad, Hercules CA, United States) was used with run parameters of 3.4 V/cm (113.3V) for 23.5 h, with switch times changed from 0.2 to 0.8 s. Tank buffer temperature was maintained at 14°C for experiment entirety. The gel was stained with a 3X GelRed solution for 1.5 h followed by a 30 min de-staining with distilled water and analyzed using Bio-Rad ChemiDoc XRS gel imaging system and Quantity One software version 4.6.5.

### Phylogenetic Tree Construction

Phylogenetic analyses of the SU57 genome and other T1-like phages were conducted using alignments of complete nucleotide sequences as well as predicted amino acid sequences of selected genes. Sequences and references for whole genomes and selected proteins (major capsid protein, terminase large subunit, tail fiber protein, and portal protein) were obtained from the NCBI genome databases. The genome nucleotide sequence of SU57 was aligned to genomes showing E-values = 0 in discontinuous MegaBLAST searches against the nucleotide collection database limited to *Caudovirales* phages, with the addition of the genomes of phage T1, TLS, and F20. Phage genomes from the clinical samples reported by Pacífico et al. (2019) were excluded for being too similar between themselves and as they were all 86–88% identical (E-value = 0) to phages vB_Ecos_CEB_EC3a, Rtp, and 2725-N35 (See Supplementary Table 2 for accession numbers to genome sequences).

Each inferred amino acid sequence of the four structural proteins of SU57 was aligned to the 18 most similar amino acid sequences found by Blastx searches against the nr protein database, and complemented with the corresponding protein sequences of phages T1 and TLS. Alignments were made in ClustalW with default settings embedded in the Mega-X software and in MAUVE version 20150226 with default settings (Mauve^6^; Thompson et al., 1994; Darling et al., 2004; Kumar et al., 2018). Neighbor-joining phylogenetic trees were constructed using Mega-X default settings. Node confidence was evaluated using bootstrap testing based on 500 random re-samplings.

## Results and Discussion

### SU57 Phage Plaque and Virion Morphology

**FIGURE 1:**
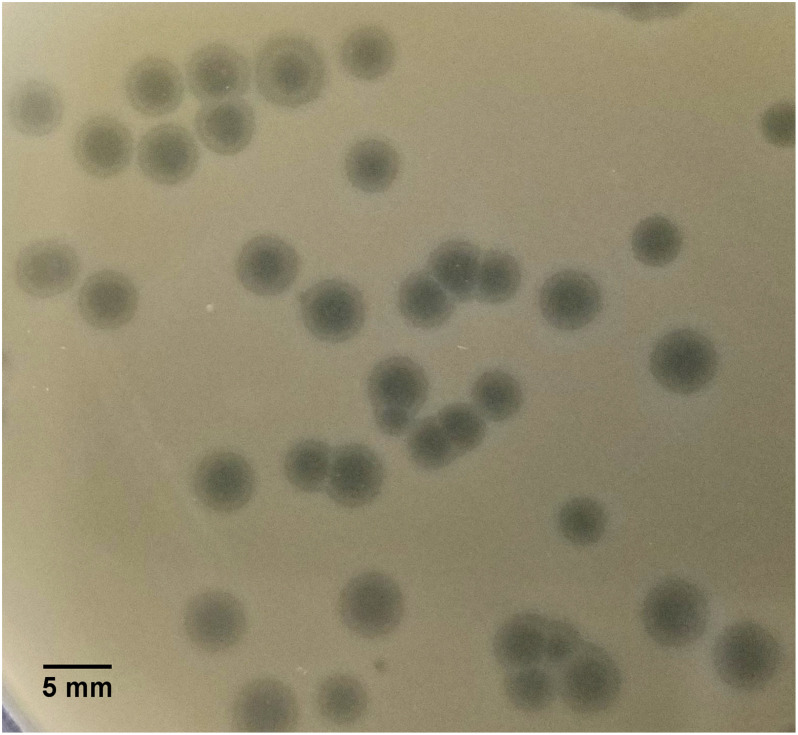
Plaque morphology of SU57. Phages were cultured on ECOR57, from the *Escherichia coli* reference collection, forming 4 mm plaques (4.1 ± 0.7 mm in diameter) with a clear center surrounded by a halo.

**FIGURE 2:**
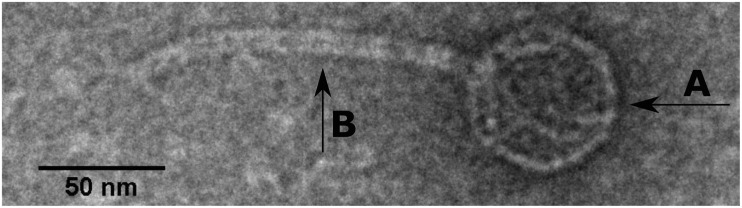
Transmission electron microscope micrograph of negatively stained phage SU57 at 30,000 × magnification. SU57 has **(A)** a head diameter of approximately 54 nm (54 ± 4 nm), **(B)** a flexible non-contractile tail approximately 148 nm long (148 ± 8 nm).

SU57 forms transparent, circular plaques with halos approximately 4 mm in diameter (4.1 ± 0.7 mm) upon a lawn of its host bacteria ECOR57 (Figure 1). This is in accordance with what has been observed for other T1-like phage infections (Feiner and Hill, 1966; Roberts et al., 2004; Niu et al., 2014; Hamdi et al., 2017). TEM confirms that SU57 is a phage belonging to the order *Caudovirales* with a head diameter of 54 nm (54 ± 4 nm) and a flexible, non-contractile tail of 148 nm (148 ± 8 nm) (Figures 2A,B). This type of phage used to be classified as belonging to the *Siphoviridae* family, but since the introduction of new phage families, phages can no longer be classified as belonging to a particular family based on micrographs only. SU57 is however comparable in head-to-tail proportions (the head diameter being approximately 36% of the tail length; Figures 2A,B) to T1-like phages like vB_EcoS_Rogue1 and vB_EcoS_ACG-M12 (Chibeu et al., 2012; Kropinski et al., 2012).

### SU57 Phage Infection Linetics

**FIGURE 3:**
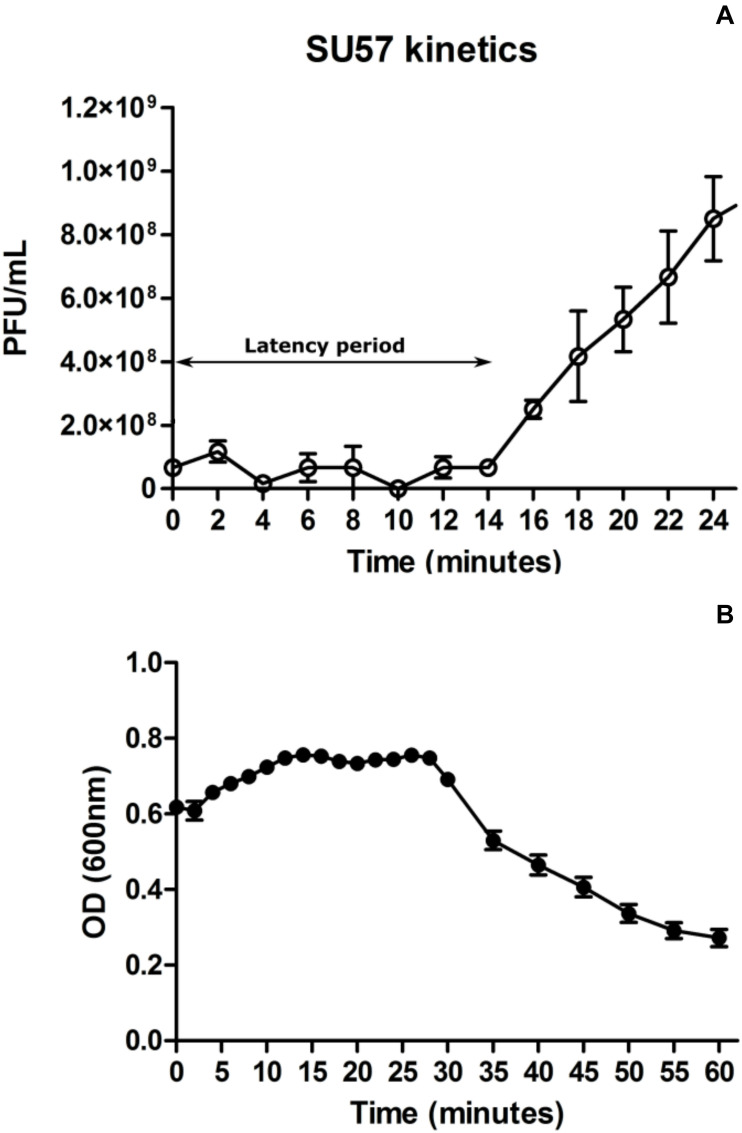
One step growth curve of phage SU57 infecting the ECOR57 *Escherichia coli* strain at a multiplicity of infection (MOI) of 0.14 **(A)**. Growth curve of SU57 using the modified standard protocol (see text for details). The latency period for SU57 is 14 min with a burst size of 13 plaque forming units (PFU)/cell. Data points represents the mean of three independent experiments and error bars represents the standard error (SEM) of each run. **(B)**. ECOR57 bacterial lysis following an SU57 infection at a MOI of 0.14 represented as a change in optical density at 600 nm. Data points represents the mean of three independent experiments and error bars represents SEM of each run.

At a MOI of 0.14, SU57 has a short latency period of 14 min (determined as the time from addition of phages to significant rise in phage titer) and a burst size of 13 PFU/cell (Figure 3A and Supplementary Figure 1). The adsorption rate constant 4 min post infection was determined to be 8.5 × 10^–10^ mL × min^–1^. As shown in Figure 3B, a drastic drop in OD_600_ was however seen 30 min after infection by SU57, suggesting a more substantial phage induced bacterial lysis. Using optical density readings to determine SU57’s latency period, interpreting the sudden decrease in OD_600_ with the occurrence of extensive phage induced bacterial lysis, the decrease was however detected about 30 min post infection with SU57 (Figure 3B). The discrepancy observed, between using the one-step growth curve and optical density to determine the latency period, can be attributed to the phage titer used for infection. The phage titer used, resulting in a MOI 0.14, was too small to cause an observable decrease in OD_600_ 14 min post infection, since few cells got infected and since the burst size is quite small. The drop in OD_600_ seen could therefore be the result of the second round of infection by the newly formed phage progeny. It is important to note that there can be limitations to using the one-step growth curve methodology to determine fast phage kinetics or phages exhibiting a small burst size. Fast phages could experience multiple rounds of infection, from the initial standing period post infection followed by the removal of non-adsorbed phages to experimental time zero, thereby not providing a real time representation of fast phage kinetics. A small burst size can also pass unnoticed and be hard to detect, at least with turbidimetry. On the other hand, a high MOI may lead to spurious results while it cannot be ruled out that the size of the burst can be affected by more than one phage infecting a bacterium, i.e., the bacterium might be able to produce more phages if infected by several phages. The present results are however in contrast to the previous findings on SU57, which showed about the same latency period, but a much larger burst size of 183 PFU/cell (Khan Mirzaei and Nilsson, 2015). A possible explanation for this difference is the variance arising from the methods applied on bacteria and phage counts. In this study, the burst size was calculated as the ratio between the phage progeny titer and the phage adsorbed titer. Whereas, in the previous study the burst size was calculated as the difference between the free phage titer and the un-adsorbed titer divided by the initial phage titer. It is also possible that with a higher MOI of five, the bacteria was infected by more than one phage, resulting in a sustained infection and a larger burst size (Abedon, 2012b). The concentration of divalent ions such as Mg^2+^ or Ca^2+^ in the growth media could have also contributed to the large difference in burst size, as they have been shown to increase adsorption rate in many phages (Chhibber et al., 2014). In both these experiments however, the divalent ion concentration was not accounted for.

Phage virulence is dependent on multiple factors, including their ability to mount a productive infection within their host (Brüssow et al., 2004). As seen with phages T1, vB_EcoS_CEB_EC3a, and SH6, a short latency period is mainly caused by a fast adsorption rate and a relatively small burst size (Roberts et al., 2004; Hamdi et al., 2017; Oliveira et al., 2018). It could be hypothesized that a short latency period and small burst size is a trade-off which increases the chances of phage survival under certain ecological conditions (Abedon et al., 2001; Abedon, 2016). Recent characterization of the T1-like phages vB_EcoS-95 and vB_EcoS-IME253 however shows a very short latency period of 4 and 5 min combined with large burst sizes of 115 PFU/cell and 186 PFU/cell, respectively (Li et al., 2019; Topka et al., 2019). Latency periods and burst sizes are dependent on the status of their hosts and the ability to synthesize their proteins (Helmstetter and Cooper, 1968; You et al., 2002). In the case of a vB_EcoS-IME253 infection, a single *E. coli* cell would have to synthesize 31,000 base pairs of DNA per second as the genome size of this phage is approximately 50,000 base pairs. In addition to this, the cell also needs to transcribe and translate all the structural genes for assembly of the 186 phage particles. It seems more likely that a very short latency time is accompanied by a small burst size both from a phage adaptation point of view and as a consequence of a cell’s capacity of synthesizing DNA and proteins.

### SU57 Genome Characterization

Assembly of the 30,582 reads (average fragmented size 2,000 base pairs) from the sequencing of the SU57 genome resulted in a 46,150 base pair contig of a unique sequence, and the genome size was confirmed by a second assembly using SPAdes on the same dataset. Preliminary BLASTn analyses showed that SU57 was closely related to T1-like phages of the *Drexlerviridae* family (formerly part of the *Siphoviridae*) (Supplementary Table 2) (German et al., 2006; Niu et al., 2014; Adriaenssens et al., 2020). As T1-like phage genomes are circularly permuted, an exact length of the entire genome could not be determined, but the 46,150 bp sequence should contain the terminal repeat region. This was also reflected in the search for the genome ends. PhageTerm reported several possible ends but one of these was located at an abrupt change in sequence read frequency and, upon comparison with the genomes of phage T1 and the direction of putative transcripts in the SU57 genome, was determined to be the most likely end. This means that the terminal repeat in the beginning of the SU57 genome is located in a UTR followed by many small ORFs coding for hypothetical proteins, and the genome ends in a similar fashion with many short ORFs and a UTR. The *pac* site of SU57 was not identified but is located in the corresponding region in the genome of T1 as well as in other T1-like phages.

**FIGURE 4:**
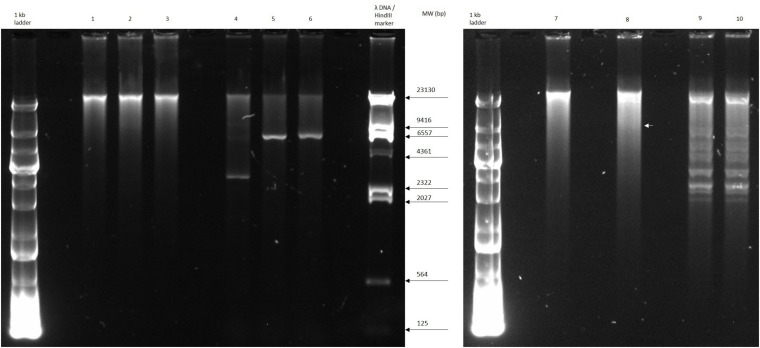
Restriction digest analyses of phage SU57. Lanes 1 and 7, undigested SU57 DNA; lane 2, SU57 DNA digested with *Sac*I; lane 3, SU57 DNA digested with *Apa*I; lanes 4–6, controls used for digestion: undigested Pet26b(+) plasmid, Pet26b(+) digested with *Sac*I, and *Apa*I, respectively; lane 8, SU57 DNA double digested with *Sac*I and *Apa*I; lanes 9 and 10, *Xho*I-digested SU57 DNA. Lane 10 contains DNA that was digested, heated to 80°C and then chilled on ice before gel electrophoresis. A faint band (white arrow) is marked in lane 8 after the double digest.

**FIGURE 5:**
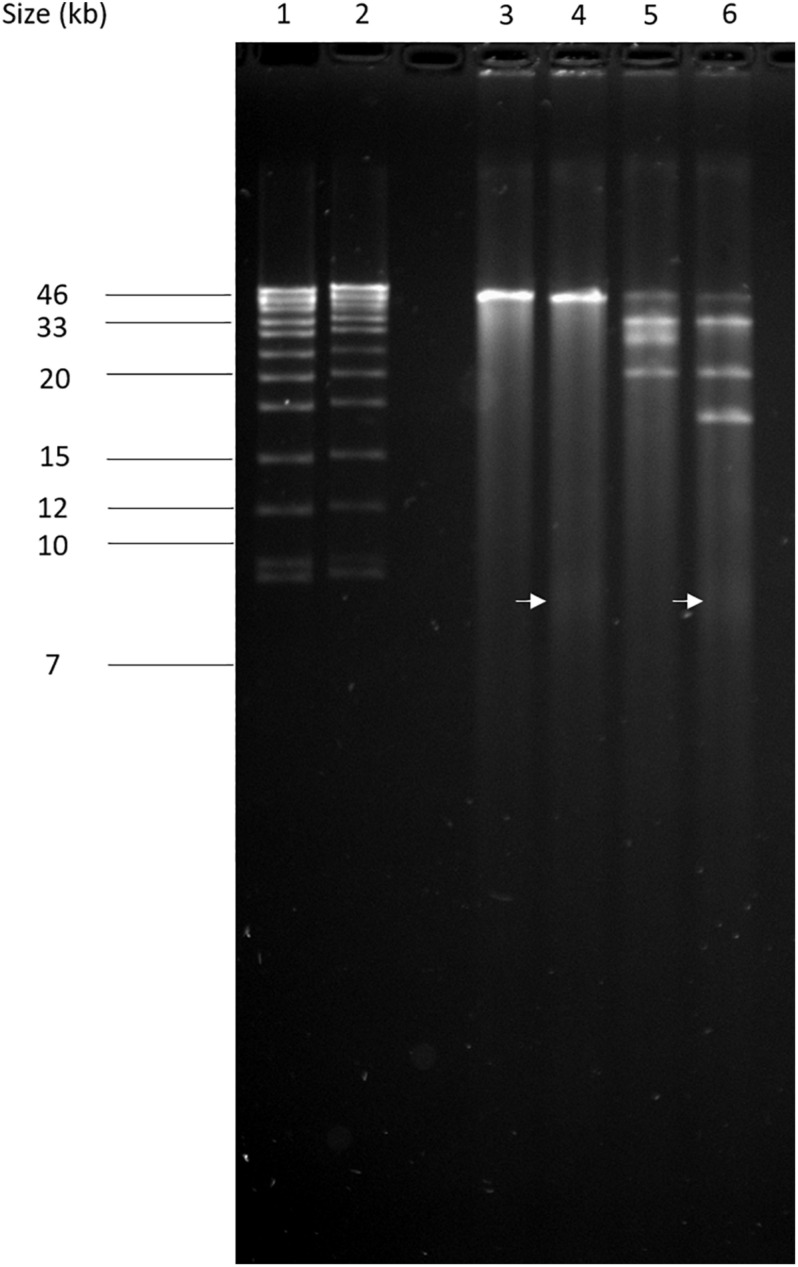
Pulse field-gel analyses of restriction enzyme-digested SU57 phage DNA. Lanes 1 and 2, pre-digested λ DNA standard as a positive control and a molecular weight (in kb) marker; lane 3, undigested SU57 DNA; lane 4, SU57 DNA digested with *Sac*I; lane 5, SU57 DNA digested with *Apa*I; lane 6, SU57 DNA double digested with *Sac*I and *Apa*I. A faint band with a molecular weight less than 8.3 kb (white arrows) can be seen in the *Sac*I single and double digested lanes.

The circular permuted nature of SU57 was further supported by restriction analyses (Figure 4). Undigested SU57 phage DNA appeared as a single band with a smear. When using unique cutting restriction enzymes (*Sac*I cutting at position 40,723 and *Apa*I cutting at position 26,170), similar banding patterns to uncut DNA were observed. Upon restriction digest, a DNA smear may be indicative of a circular permuted genome as cutting close to *pac* site can produce similarly long fragments (Summer et al., 2006). Additionally, a smear can indicate random site packaging as well as the possibility of initiation occurring at multiple *pac* sites (Summer et al., 2006). A double digest using *Sac*I and *Apa*I produced two bands (molecular weights >20,000 and >10,000 bp, respectively). SU57 genome has ten recognition sites for *Xho*I and digestion gave the pattern expected from a covalently close circular molecule. Additionally, no *cos* site was found when SU57 DNA was digested with *Xho*I, heated to 80°C, and rapidly cooled before gel electrophoresis. Genomes that have single-stranded cohesive ends and are enzyme digested will produce fragments with *cos* sites. Gradual temperature cooling will allow these fragments to join together and appear as a larger band on a gel (Paul et al., 2005; Cheepudom et al., 2015). No change in the *Xho*I restriction pattern of SU57 resulting from different cooling processes (gradual or rapid) was observed, excluding the genomic presence of *cos* sites. A sub-molar fraction indicative of a *pac* site was also not seen. Additional pulse field gel electrophoresis analyses were done on the restriction enzyme-digested SU57 DNA to determine if separate the bands could be observed from the DNA smear (Figure 5). Undigested SU57 phage DNA appeared as a single sharp band (approximately 46 kb), with a larger smear size compared to the digested DNA (Figure 5, lanes 4–6). *Sac*I digested DNA produced a smaller smear with a sharp band, similar to the uncut DNA, as well as a faint broad band below the 8.3 kb size marker (white arrows). *Apa*I digested SU57 DNA produced four distinct bands (approximately 46, 33, 30, and 20 kb). *Sac*I and *Apa*I double digested SU57 produced a smear and similar banding patterns to the single enzyme digested DNA with the addition of a band approximately 17 kb and the removal of the 30 kb band. Since *Sac*I and *Apa*I are unique cutting restriction enzymes, the presence of multiple bands is indicative of SU57’s circular permuted nature. Phages with a circular permuted genome reloads from its cutting site and packs headful before its DNA is cut again. As this is repeated several times, the phage DNA will be a mix of same length molecules with the restriction cutting site at different locations (Garneau et al., 2017). These results are similar to analyses of phages that utilize a headful packing mechanism and are circularly permuted: P22, Sf6, and φHSIC (Casjens et al., 2004; Paul et al., 2005). Because the ends of these genomes occur at different places on different molecules, restriction digests are not capable of showing the exact locations of the ends, and as such produce restriction analyses for a circular genome (Casjens et al., 2004; Paul et al., 2005).

The SU57 genome has a GC content of 43.96% and lacks most *E. coli* restriction enzyme cutting sites. No site was found for the *E. coli* B and K enzymes *Eco*BI or *Eco*KI, which are not present in phage T1 either (German et al., 2006), and the only *E. coli* derived restriction enzymes that presumably would digest the genome would be *Eco*31I, *Eco*57MI (*Eco*57I), and *Eco*P15I.

**FIGURE 6:**
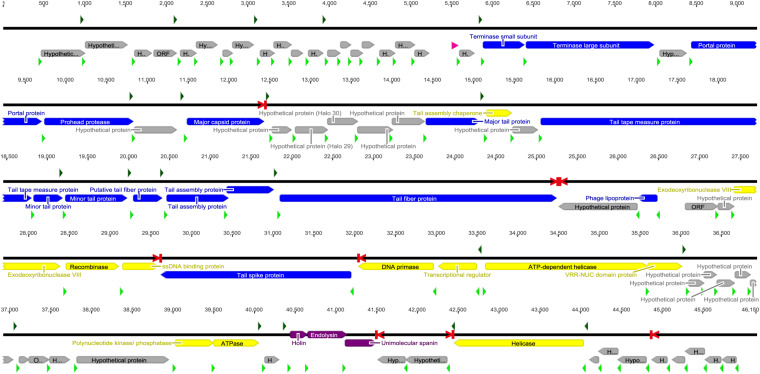
The SU57 genome was assembled using Canu and SPAdes and annotated in Geneious 6.1.8. Transcripts were identified by Softberry BPROM as starting at bacterial σ^70^ promoter regions (green arrows) and ending at rho-independent transcription terminators (red > |) identified with ARNold. Open reading frames (ORFs) were predicted using GLIMMER3 and annotated by similarities to proteins found in Blastx searches of the nr protein database at the NCBI website. Putative ribosomal binding sites (RBSs, light green arrows) were identified in untranslated regions upstream of ORFs. A gene was only considered to encode a protein if an RBS was found. Blue bars represent proteins associated with phage structure, yellow the proteins involved in DNA metabolism, purple the proteins associated with lysis, and gray hypothetical proteins or ORFs. The tRNA gene (pink) was found using ARAGORN 1.2.38. See section “Materials and Methods” for references to the computer programs used.

Further analyses of the genome revealed 20 transcripts initiated at hypothetical *E. coli* σ^70^ promoters, eight putative rho-independent transcription terminator sites, 80 ORFs, and a putative tRNA gene (Figure 6). The order of transcripts and the order and size of ORFs within transcripts was similar to other T1-like phages. Of the 80 ORFs, 29 were considered to encode proteins with a known function as judged by Blastx similarities to proteins in GenBank. These 29 coding regions are commonly found in other T1-like phages, and especially the inferred structural proteins show great similarity to structural proteins of other T1-like phages (Supplementary Table 1). Interestingly, a tail spike gene is located in a transcript on the bottom strand after the recombination block of genes. The beginning of the gene 31,958 – 31,194 is present in many phage genomes, and is closely related to phage DTL, whereas the region 31,194–31,500 is unique to SU57 and cannot be found in other phages nor in gammaproteobacteria genomes. The ending sequence 31,500–29,625 is only similar to one other phage gene, coding for a hypothetical protein in the *Escherichia* phage 2725-N35 genome. In addition, the only difference between the two genome assemblies was within this gene, an extra “T” at 31,225 in the genome assembled with Canu as compared to the resulting genome from the SPAdes assembly. Hypothetically, this indicates a site for regular recombination, but there is however a putative rho-independent terminator located before the tail spike gene and it is uncertain if the gene gets transcribed at all.

The genome of SU57 does not seem to contain a gene encoding for phage RNA polymerase, and the transcription of phage genes is most likely carried out entirely by the machinery of the bacterium. Translation of the AGA codon, quite rare in the *E. coli* genome, is possibly facilitated by a phage encoded tRNA^Arg^ gene. The genome however contains many genes encoding proteins involved in DNA replication e.g., a DNA primase and an ssDNA binding protein. The origin of replication (resembling an *E. coli* oriC site) was on the other hand not found.

### Phylogenetic Analyses

**FIGURE 7:**
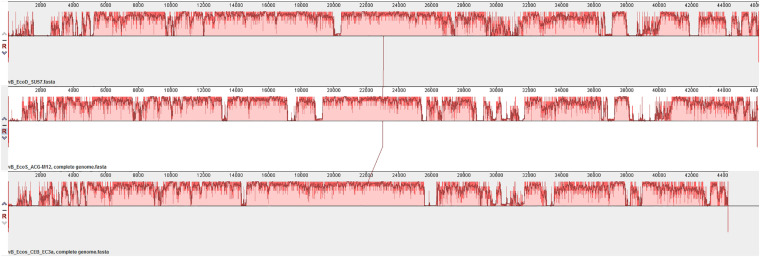
Multiple genome alignment of SU57 and closely related T1-like genomes, vB_EcoS_ACG-M12 and vB_Ecos_CEB_EC3a, using MAUVE. Bar heights represent the average level of conservation within each region of the genome sequences. White regions represent fragments that have not been aligned or have sequence specific components.

**FIGURE 8:**
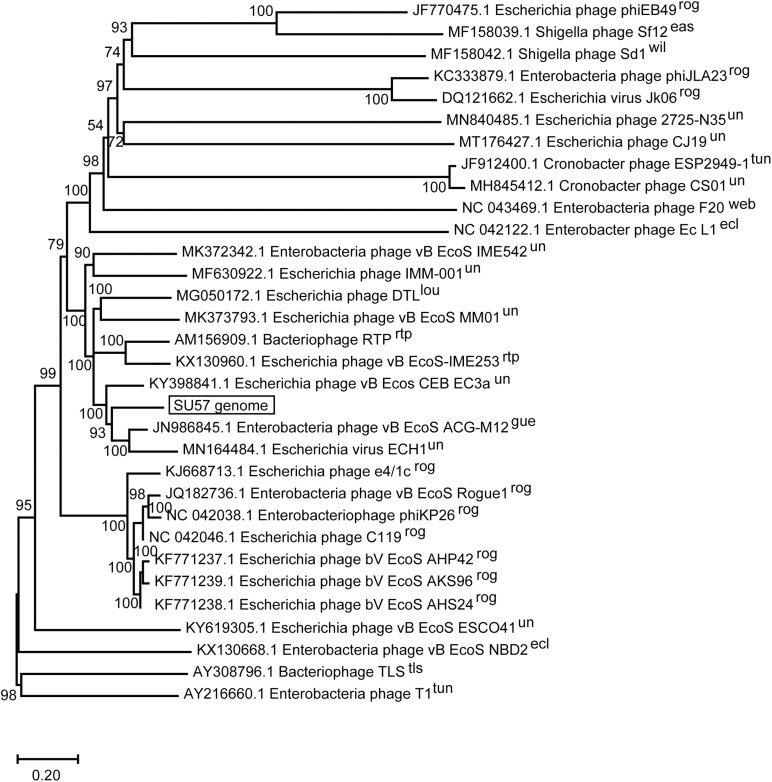
Neighbor-joining phylogenetic tree of phage SU57 whole genome nucleotide sequence in relation to other T1-like phages. The sequence of SU57 and genome sequences showing an E-value of 0 in discontinuous megablast searches against the nucleotide collection database at the NCBI website was aligned in ClustalW together with the nucleotide sequence of phage T1 and TLS. The tree was constructed using Mega-X. Shown at the nodes are bootstrap values which were calculated based on 500 replicates. The bar represents the number of nucleotide substitutions per site. Taxonomic classification to genera is indicated after the phage name, where rog, Rogunavirus; eas, Eastlansingvirus; wil, Wilsonroadvirus; tun, Tunavirus; web, Webervirus; lou, Loudonvirus; rtp, Rtpvirus; gue, Guelphvirus; rog, Rogunavirus; ecl, Eclunavirus; tls, Tlsvirus; un, unclassified.

The SU57 genome is similar to four phages in particular. Needleman-Wunsch global pairwise alignments of the nucleotide sequences of SU57 and these genomes showed that, apart from the small genes at both ends of the genome encoding hypothetical proteins, SU57 has most of its genes in common with the *Escherichia* phages vB_Ecos_CEB_EC3a and vB_EcoS_ACG-M12, both showing a nucleotide identity of 72% over their complete genomes (Figure 7), and phage DTL showing 55% identity over its entire genome. Phage ECH1 is also phylogenetically closely related but has differentiated to be only 49% identical (Altschul et al., 1997; Chibeu et al., 2012; Halter and Zahn, 2018; Oliveira et al., 2018). The clustering in a phylogenetic analysis based on whole genome nucleotide sequences reflects both the number of similar genes and the degree of similarity of those genes, and the analysis of these T1-like phages does not discriminate between these. These phages may thus share some but not all genes with SU57 (Figure 8). Although the taxonomic position to genera of ECH1 and vB_Ecos_CEB_EC3a is not established, and other members of the cluster classified into different genera (Guelphvirus and Rtpvirus, respectively), SU57 can at least be determined to belong to the subfamily *Braunvirinae* within *Drexlerviridae*. References to the T1-like *Drexlerviridae* phages in the phylogenetic analyses and their accession numbers can be found in Supplementary Table 2.

**FIGURE 9:**
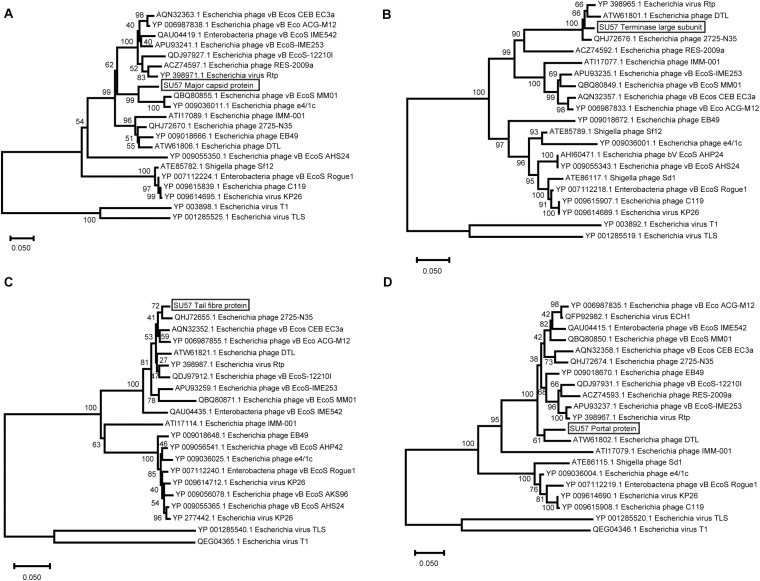
Neighbor-Joining phylogenetic tree representing the relationship between structural proteins of phage SU57, the 18 most similar proteins found by Blastx searches against the nr protein database at the NCBI website, and the corresponding proteins from phage T1 and TLS. The trees were constructed using Mega-X after performing alignments of the protein residues with ClustalW. Bootstrap values representing 500 re-samplings are presented at the nodes, and the bar represents the number of amino acid substitutions per site. **(A)** Major capsid protein. **(B)** Terminase large subunit. **(C)** Tail fiber protein. **(D)** Portal protein.

Phylogenetic analyses were also carried out on a selection of inferred amino acid sequences from four genes encoding structural proteins. All of these proteins are presumably evolutionary conserved, as indicated by small sequence differences between different phages. The major capsid protein has not differentiated much among the analyzed proteins and varies between 90% identical residues in phage e4/1c (accession # YP_009036011.1) down to 51% identical residues in the capsid protein of the most distant relative in the phylogenetic tree, phage T1 (accession # YP_003898.1) (Figure 9A). This means that the differentiation of the major capsid gene between T1-like phages in general is relatively small. The large terminase subunit protein is also less differentiated in the close relatives to SU57 as the large terminase protein of phage Rtp shows 97% identical residues (accession # YP_398965.1). Even the most distantly related large terminase phage proteins in the analysis are 98% the same size although the percentage of identical residues falls to 54% identical residues in phage T1 (accession # YP_003892.1) compared to SU57 (Figure 9B). The large tail fiber protein is the largest protein encoded by the SU57 genome. One would expect specificity for various host cell surface receptors to be reflected in the variation of tail fibers, but this protein is surprisingly similar among the cluster of phages most closely related to the SU57 tail fiber protein with 96–98% identical residues (Figure 9C). Proteins from the more distantly related group of phages are however not particularly similar. Phage T1’s tail fiber is for instance 56% identical (accession # QEG04365.1), with the C-terminal end being partly completely different. While it is known that phage T1 and TLS bind to different cell surface receptors, and considering the topology of the tree and the distribution of the variation, it can be hypothesized that these phages can be divided into at least four groups of cell surface receptors. The portal protein of phage vB_Ecos_CEB_EC3a is 94% similar to the SU57 portal protein and shows variation only in the C-terminal end (accession # AQN32358.1). The portal protein of phage T1 is the most distantly related protein included in the analysis, but still somewhat similar to the SU57 portal protein with 54% identical residues (accession # QEG04346.1) (Figure 9D).

## Data Availability Statement

The datasets presentedin this study can be found in online repositories. The names of the repository/repositories and accession number(s) can be found below: https://www.ncbi.nlm.nih.gov/nuccore/MT511058.

## Author Contributions

SK, CC, and AN contributed to study design and conception and wrote the manuscript. CC purified and extracted phage DNA. SK and FS performed the experiments. SK, FS, and AN performed the bioinformatics analyses of the genome. SK and AN performed the phylogenetic analyses. All authors contributed to the article and approved the submitted version.

## Conflict of Interest

The authors declare that the research was conducted in the absence of any commercial or financial relationships that could be construed as a potential conflict of interest.

## Footnotes

1 https://galaxy.pasteur.fr/

2 https://www.geneious.com

3 http://rssf.i2bc.paris-saclay.fr/toolbox/arnold/index.php (accessed November 5, 2019).

4 http://www.bioinformatics.nl/cgi-bin/emboss/geecee?_pref_hide_optional=0 (accessed May 13, 2020).

5 https://www.bioinformatics.nl/cgi-bin/emboss/restrict (accessed May 13, 2020).

6 http://darlinglab.org/mauve/mauve.html (accessed July 1, 2020).

## Supplementary Material

Supplementary Figure 1 One step growth curve of phage SU57 over 60 min.

Supplementary Table 1 General features of presumed ORFs from phage SU57.

Supplementary Table 2 T1-like *Drexlerviridae* (*Siphoviridae*) phages used for phylogenetic analyses.
