# Supplementary figures and images for: Infection Kinetics and Phylogenetic Analysis of vB_EcoD_SU57, a Virulent T1-Like Drexlerviridae Coliphage
Source: Front Microbiol. 2020 Nov 16;11:565556. doi: 10.3389/fmicb.2020.565556 (PMC7718038; doi:10.3389/fmicb.2020.565556)

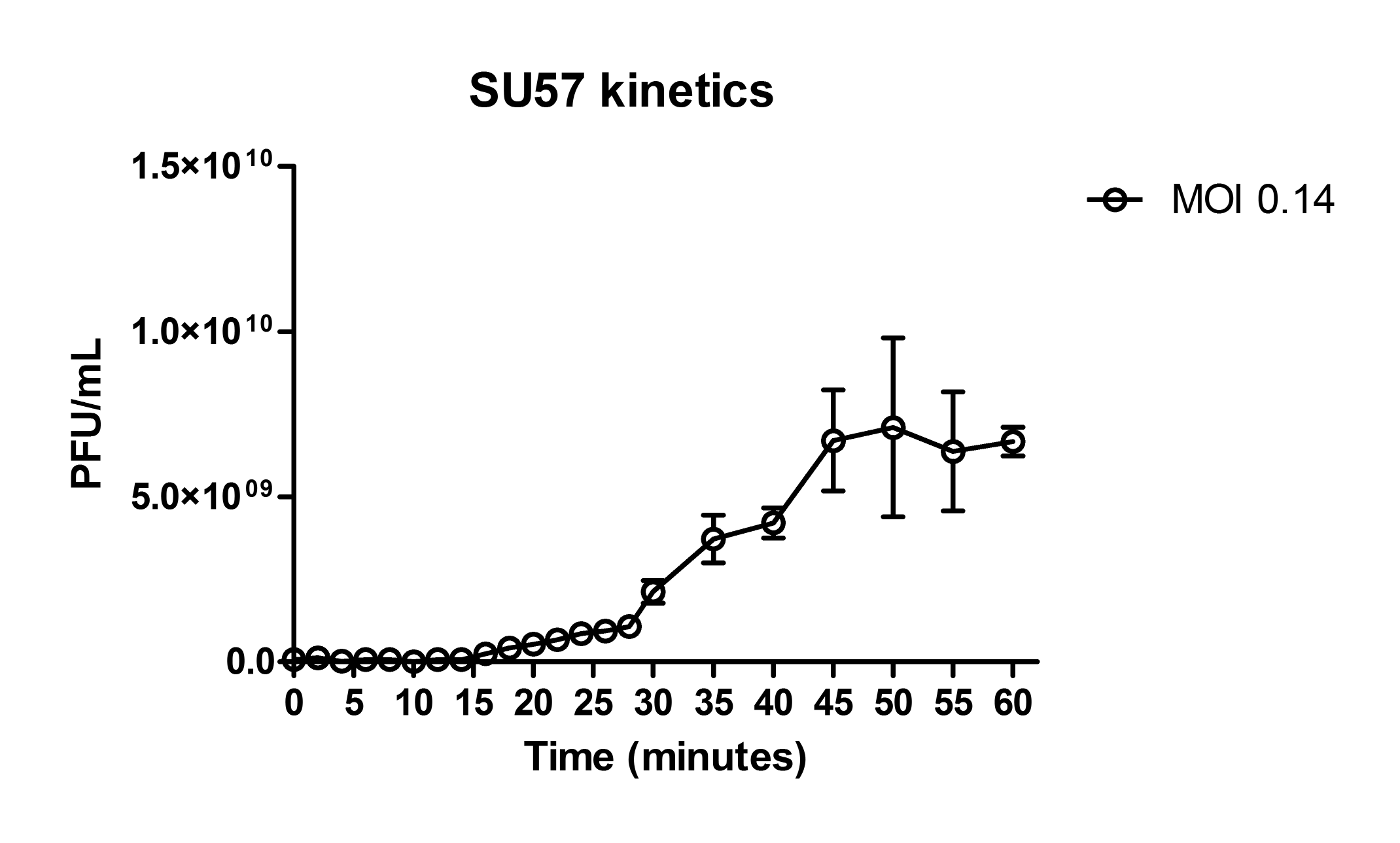

Supplement: Supplementary Figure 1 — One step growth curve of phage SU57 over 60 min. [file Image_1.TIF]
