# Supplementary material for: Infection Kinetics and Phylogenetic Analysis of vB_EcoD_SU57, a Virulent T1-Like Drexlerviridae Coliphage
Source: Front Microbiol. 2020 Nov 16;11:565556. doi: 10.3389/fmicb.2020.565556 (PMC7718038; doi:10.3389/fmicb.2020.565556)
Supplement: Supplementary Table 1 — General features of presumed ORFs from phage SU57. [file Table_1.DOCX]

Supplementary Table 1. General features and functions of presumed ORFs from phage SU57. ORF lengths are represented as nucleotides (nt) and the coding strand of DNA where the ORF is located as either top (T) or bottom (B) strand. Identity is the percentage of identical residues in the most similar protein found in the nr NCBI database. Note: Function is labeled as ORF if there was no ribosomal binding site (RBS) located upstream of the ORF start codon. ORFs 19 and 73 are labeled as hypothetical protein due to the presence of RBS sites, but no matches were found in the NCBI nr database.

| ORF | Length (nt) | Strand | Function | Query Coverage (%) | E-value | Identity (%) | Accession number |
| --- | --- | --- | --- | --- | --- | --- | --- |
| 1 | 543 | T | Hypothetical protein | 99 | 2.00E-69 | 60.44 | [ATI17050.1](https://www.ncbi.nlm.nih.gov/protein/ATI17050.1?report=genbank&log$=prottop&blast_rank=1&RID=VDMT3TWZ014) |
| 2 | 525 | T | Hypothetical protein | 99 | 1.00E-104 | 83.91 | [APU93221.1](https://www.ncbi.nlm.nih.gov/protein/APU93221.1?report=genbank&log$=prottop&blast_rank=1&RID=VDMX3Z0J015) |
| 3 | 219 | T | Hypothetical protein | 98 | 1.00E-43 | 94.44 | [AQY55258.1](https://www.ncbi.nlm.nih.gov/protein/AQY55258.1?report=genbank&log$=prottop&blast_rank=1&RID=VDMZTZTE014) |
| 4 | 288 | T | ORF |  |  |  |  |
| 5 | 204 | T | Hypothetical protein | 98 | 1.00E-17 | 79.41 | [QHJ72689.1](https://www.ncbi.nlm.nih.gov/protein/QHJ72689.1?report=genbank&log$=prottop&blast_rank=1&RID=BT0WSFSB014) |
| 6 | 264 | T | Hypothetical protein | 97 | 7.00E-57 | 93.02 | [QHJ72688.1](https://www.ncbi.nlm.nih.gov/protein/QHJ72688.1?report=genbank&log$=prottop&blast_rank=1&RID=A6JGB1CN014) |
| 7 | 126 | T | Hypothetical protein | 97 | 3.00E-20 | 85.37 | [YP_009614678.1](https://www.ncbi.nlm.nih.gov/protein/YP_009614678.1?report=genbank&log$=prottop&blast_rank=1&RID=BUH4J6B8014) |
| 8 | 261 | T | Hypothetical protein | 98 | 3.00E-54 | 93.02 | [QDJ97942.1](https://www.ncbi.nlm.nih.gov/protein/QDJ97942.1?report=genbank&log$=prottop&blast_rank=1&RID=BT386EHZ016) |
| 9 | 186 | T | Hypothetical protein | 90 | 1.00E-20 | 71.43 | [APU93223.1](https://www.ncbi.nlm.nih.gov/protein/APU93223.1?report=genbank&log$=prottop&blast_rank=1&RID=VFV444GG014) |
| 10 | 219 | T | Hypothetical protein | 98 | 2.00E-23 | 64.56 | [YP_006987823.1](https://www.ncbi.nlm.nih.gov/protein/YP_006987823.1?report=genbank&log$=prottop&blast_rank=2&RID=VFV7DYNV014) |
| 11 | 144 | T | Hypothetical protein | 93 | 9.00E-24 | 93.33 | [QAU04406.1](https://www.ncbi.nlm.nih.gov/protein/QAU04406.1?report=genbank&log$=prottop&blast_rank=1&RID=VFVA430Z014) |
| 12 | 183 | T | Hypothetical protein | 95 | 2.00E-33 | 96.55 | [QAU04407.1](https://www.ncbi.nlm.nih.gov/protein/QAU04407.1?report=genbank&log$=prottop&blast_rank=1&RID=VFVDBVK5014) |
| 13 | 162 | T | Hypothetical protein | 96 | 3.00E-18 | 73.08 | [QBQ80844.1](https://www.ncbi.nlm.nih.gov/protein/QBQ80844.1?report=genbank&log$=prottop&blast_rank=2&RID=VFVH7DSC015) |
| 14 | 132 | T | Hypothetical protein | 97 | 1.00E-16 | 69.77 | [ATI17064.1](https://www.ncbi.nlm.nih.gov/protein/ATI17064.1?report=genbank&log$=prottop&blast_rank=1&RID=FM9V504V016) |
| 15 | 153 | T | Hypothetical protein | 86 | 6.00E-13 | 59.09 | [AQN32416.1](https://www.ncbi.nlm.nih.gov/protein/AQN32416.1?report=genbank&log$=prottop&blast_rank=1&RID=VFVWWD2Z015) |
| 16 | 153 | T | Hypothetical protein | 98.6 | 9.00E-29 | 90.00 | [APU93229.1](https://www.ncbi.nlm.nih.gov/protein/APU93229.1?report=genbank&log$=prottop&blast_rank=1&RID=VFW32REF014) |
| 17 | 198 | T | Hypothetical protein | 98 | 8.00E-35 | 86.57 | [QDJ97936.1](https://www.ncbi.nlm.nih.gov/protein/QDJ97936.1?report=genbank&log$=prottop&blast_rank=1&RID=BT11H2SK016) |
| 18 | 240 | T | Hypothetical protein | 98 | 2.00E-48 | 92.41 | [AQN32399.1](https://www.ncbi.nlm.nih.gov/protein/AQN32399.1?report=genbank&log$=prottop&blast_rank=1&RID=VFW9A50G014) |
| 19 | 183 | T | Hypothetical protein |  |  |  |  |
| 20 | 183 | T | Hypothetical protein | 98 | 2.00E-36 | 100.00 | [QBQ80847.1](https://www.ncbi.nlm.nih.gov/protein/QBQ80847.1?report=genbank&log$=prottop&blast_rank=1&RID=VFWGDPN7014) |
| 21 | 507 | T | Terminase small subunit | 99 | 6.00E-117 | 98.21 | [APU93234.1](https://www.ncbi.nlm.nih.gov/protein/APU93234.1?report=genbank&log$=prottop&blast_rank=1&RID=VFWJSGTC014) |
| 22 | 1,572 | T | Terminase large subunit | 99 | 0 | 95.98 | [ATW61801.1](https://www.ncbi.nlm.nih.gov/protein/ATW61801.1?report=genbank&log$=prottop&blast_rank=2&RID=VFWP6C1Y015) |
| 23 | 333 | T | Hypothetical protein | 61 | 3.00E-24 | 64.71 | [YP_009018671.1](https://www.ncbi.nlm.nih.gov/protein/YP_009018671.1?report=genbank&log$=prottop&blast_rank=1&RID=FMAWX50X014) |
| 24 | 1,263 | T | Portal protein | 99 | 0.0 | 92.14 | [YP_009795715.1](https://www.ncbi.nlm.nih.gov/protein/YP_009795715.1?report=genbank&log$=prottop&blast_rank=2&RID=C7KSGNME014) |
| 25 | 1,089 | T | Prohead protease | 99 | 0 | 92.82 | [ATW61803.1](https://www.ncbi.nlm.nih.gov/protein/ATW61803.1?report=genbank&log$=prottop&blast_rank=3&RID=VFX57TZZ015) |
| 26 | 522 | T | Hypothetical protein | 98 | 6.00E-93 | 99.42 | [QHJ72672.1](https://www.ncbi.nlm.nih.gov/protein/QHJ72672.1?report=genbank&log$=prottop&blast_rank=1&RID=BT152RXY016) |
| 27 | 948 | T | Major capsid protein | 99 | 0.0 | 89.84 | [QBQ80855.1](https://www.ncbi.nlm.nih.gov/protein/QBQ80855.1?report=genbank&log$=prottop&blast_rank=1&RID=C7KXV9BS016) |
| 28 | 246 | T | Hypothetical protein | 98 | 1.00E-52 | 97.53 | [YP_009036012.1](https://www.ncbi.nlm.nih.gov/protein/YP_009036012.1?report=genbank&log$=prottop&blast_rank=1&RID=VFXME8UF014) |
| 29 | 402 | T | Hypothetical protein | 99 | 2.00E-89 | 97.74 | [ACZ74599.1](https://www.ncbi.nlm.nih.gov/protein/ACZ74599.1?report=genbank&log$=prottop&blast_rank=2&RID=VFXPZ7J1015) |
| 30 | 372 | T | Hypothetical protein | 99 | 1.00E-77 | 93.50 | [ACZ74600.1](https://www.ncbi.nlm.nih.gov/protein/ACZ74600.1?report=genbank&log$=prottop&blast_rank=1&RID=VFXU501T014) |
| 31 | 438 | T | Hypothetical protein | 99 | 2.00E-97 | 97.93 | [YP_006987842.1](https://www.ncbi.nlm.nih.gov/protein/YP_006987842.1?report=genbank&log$=prottop&blast_rank=1&RID=VFXYXFZ2014) |
| 32 | 402 | T | Hypothetical protein | 99 | 2.00E-88 | 94.74 | [APU93246.1](https://www.ncbi.nlm.nih.gov/protein/APU93246.1?report=genbank&log$=prottop&blast_rank=1&RID=VFY1DC5E015) |
| 33 | 657 | T | Major tail protein | 99 | 8.00E-153 | 95.87 | [YP_006987844.1](https://www.ncbi.nlm.nih.gov/protein/YP_006987844.1?report=genbank&log$=prottop&blast_rank=3&RID=C7N1UDCG016) |
| 34 | 315 | T | Tail assembly chaperone | 99 | 5.00E-67 | 98.08 | [AQN32391.1](https://www.ncbi.nlm.nih.gov/protein/AQN32391.1?report=genbank&log$=prottop&blast_rank=1&RID=VFYB0TCT014) |
| 35 | 312 | T | Hypothetical protein | 99 | 3.00E-68 | 97.09 | [APU93250.1](https://www.ncbi.nlm.nih.gov/protein/APU93250.1?report=genbank&log$=prottop&blast_rank=1&RID=VFYFVV6N015) |
| 36 | 2,979 | T | Tail tape measure protein | 99 | 0 | 96.57 | [YP_006987848.1](https://www.ncbi.nlm.nih.gov/protein/YP_006987848.1?report=genbank&log$=prottop&blast_rank=1&RID=VFYK6A01014) |
| 37 | 351 | T | Minor tail protein | 99 | 5.00E-80 | 96.55 | [YP_398982.1](https://www.ncbi.nlm.nih.gov/protein/YP_398982.1?report=genbank&log$=prottop&blast_rank=1&RID=C7NCYUCE014) |
| 38 | 756 | T | Minor tail protein | 99 | 0.0 | 98.01 | [QHJ72658.1](https://www.ncbi.nlm.nih.gov/protein/QHJ72658.1?report=genbank&log$=prottop&blast_rank=1&RID=C7NJ4AYF014) |
| 39 | 354 | T | Putatuve tail fiber protein | 86 | 1.00E-37 | 63.21 | [YP_009018651.1](https://www.ncbi.nlm.nih.gov/protein/YP_009018651.1?report=genbank&log$=prottop&blast_rank=2&RID=VFZU07FR014) |
| 40 | 759 | T | Tail assembly protein | 99 | 0 | 96.83 | [APU93256.1](https://www.ncbi.nlm.nih.gov/protein/APU93256.1?report=genbank&log$=prottop&blast_rank=1&RID=VFZYZ9P5015) |
| 41 | 573 | T | Tail assembly protein | 99 | 6.00E-122 | 97.89 | [ATW61820.1](https://www.ncbi.nlm.nih.gov/protein/ATW61820.1?report=genbank&log$=prottop&blast_rank=1&RID=VG02UAYR014) |
| 42 | 3,387 | T | Tail fiber protein | 99 | 0 | 97.70 | [QHJ72655.1](https://www.ncbi.nlm.nih.gov/protein/QHJ72655.1?report=genbank&log$=prottop&blast_rank=1&RID=C7KJ7MF1016) |
| 43 | 963 | B | Hypothetical protein | 99 | 0 | 96.56 | [YP_006987856.1](https://www.ncbi.nlm.nih.gov/protein/YP_006987856.1?report=genbank&log$=prottop&blast_rank=1&RID=VG0S7YT0015) |
| 44 | 246 | B | Phage lipoprotein | 98 | 3.00E-37 | 90.12 | [YP_006987857.1](https://www.ncbi.nlm.nih.gov/protein/YP_006987857.1?report=genbank&log$=prottop&blast_rank=2&RID=VG0V6MD6014) |
| 45 | 399 | T | ORF |  |  |  |  |
| 46 | 198 | T | Hypothetical protein | 98 | 4.00E-37 | 96.92 | [YP_006987861.1](https://www.ncbi.nlm.nih.gov/protein/YP_006987861.1?report=genbank&log$=prottop&blast_rank=1&RID=VG19RVPA014) |
| 47 | 966 | T | Exodeoxyribonuclease VIII | 99 | 0 | 92.52 | [YP_398991.1](https://www.ncbi.nlm.nih.gov/protein/YP_398991.1?report=genbank&log$=prottop&blast_rank=1&RID=VG1CMPA8015) |
| 48 | 651 | T | Recombinase | 99 | 2.00E-155 | 97.22 | [QHJ72648.1](https://www.ncbi.nlm.nih.gov/protein/QHJ72648.1?report=genbank&log$=prottop&blast_rank=1&RID=C7P0FTGB014) |
| 49 | 438 | T | ssDNA binding protein | 99 | 5.00E-78 | 86.21 | [ATI17131.1](https://www.ncbi.nlm.nih.gov/protein/ATI17131.1?report=genbank&log$=prottop&blast_rank=1&RID=C7P509WC016) |
| 50 | 2,334 | B | Tail spike protein | 18 | 4.00E-66 | 79.17 | [ATW61829.1](https://www.ncbi.nlm.nih.gov/protein/ATW61829.1?report=genbank&log$=prottop&blast_rank=3&RID=VG1ZP40N014) |
| 51 | 924 | B | DNA primase | 99 | 0 | 85.34 | [YP_006987867.1](https://www.ncbi.nlm.nih.gov/protein/YP_006987867.1?report=genbank&log$=prottop&blast_rank=1&RID=VG2EXNNG014) |
| 52 | 474 | B | Transcriptional regulator | 89 | 9.00E-99 | 96.48 | [AQN32376.1](https://www.ncbi.nlm.nih.gov/protein/AQN32376.1?report=genbank&log$=prottop&blast_rank=1&RID=VG3RRGWG014) |
| 53 | 1,995 | T | ATP-dependent helicase | 99 | 0 | 95.18 | [AQN32355.1](https://www.ncbi.nlm.nih.gov/protein/AQN32355.1?report=genbank&log$=prottop&blast_rank=2&RID=VG3VUZ66014) |
| 54 | 420 | T | VRR-NUC domain protein | 99 | 3.00E-93 | 94.24 | [AQY55311.1](https://www.ncbi.nlm.nih.gov/protein/AQY55311.1?report=genbank&log$=prottop&blast_rank=2&RID=VG3ZMFCT014) |
| 55 | 195 | T | Hypothetical protein | 98 | 4.00E-30 | 84.38 | [QBQ80884.1](https://www.ncbi.nlm.nih.gov/protein/QBQ80884.1?report=genbank&log$=prottop&blast_rank=1&RID=VG43CBM5014) |
| 56 | 159 | T | Hypothetical protein | 98 | 1.00E-24 | 90.38 | [YP_006987872.1](https://www.ncbi.nlm.nih.gov/protein/YP_006987872.1?report=genbank&log$=prottop&blast_rank=2&RID=VG45NU4H014) |
| 57 | 222 | T | Hypothetical protein | 97 | 1.00E-39 | 87.50 | [ATW61837.1](https://www.ncbi.nlm.nih.gov/protein/ATW61837.1?report=genbank&log$=prottop&blast_rank=1&RID=VD7T73C0015) |
| 58 | 198 | T | Hypothetical protein | 98 | 1.00E-35 | 96.92 | [YP_277506.1](https://www.ncbi.nlm.nih.gov/protein/YP_277506.1?report=genbank&log$=prottop&blast_rank=1&RID=VD969TZ3015) |
| 59 | 192 | T | Hypothetical protein | 98 | 6.00E-30 | 82.81 | [YP_006987875.1](https://www.ncbi.nlm.nih.gov/protein/YP_006987875.1?report=genbank&log$=prottop&blast_rank=1&RID=VD9HK6P3014) |
| 60 | 126 | T | Hypothetical protein | 97 | 1.00E-18 | 92.68 | [AQN32419.1](https://www.ncbi.nlm.nih.gov/protein/AQN32419.1?report=genbank&log$=prottop&blast_rank=1&RID=VD9N42XC014) |
| 61 | 220 | T | Hypothetical protein | 98 | 4.00E-48 | 100.00 | [YP_006987877.1](https://www.ncbi.nlm.nih.gov/protein/YP_006987877.1?report=genbank&log$=prottop&blast_rank=1&RID=A6J7P1ZP014) |
| 62 | 252 | T | Hypothetical protein | 98 | 5.00E-54 | 97.59 | [YP_006987878.1](https://www.ncbi.nlm.nih.gov/protein/YP_006987878.1?report=genbank&log$=prottop&blast_rank=1&RID=VDADM8AD014) |
| 63 | 1,131 | T | Hypothetical protein | 99 | 0 | 88.30 | [AQY55318.1](https://www.ncbi.nlm.nih.gov/protein/AQY55318.1?report=genbank&log$=prottop&blast_rank=1&RID=VDANFD4P015) |
| 64 | 474 | T | Polynucleotide kinase/ phosphatase | 99 | 5.00E-107 | 95.54 | [YP_006987881.1](https://www.ncbi.nlm.nih.gov/protein/YP_006987881.1?report=genbank&log$=prottop&blast_rank=1&RID=VDAXA8GN014) |
| 65 | 555 | T | ATPase | 83 | 1.00E-52 | 54.49 | [AQN31952.1](https://www.ncbi.nlm.nih.gov/protein/AQN31952.1?report=genbank&log$=prottop&blast_rank=4&RID=VDB9UD1H015) |
| 66 | 177 | T | Hypothetical protein | 98 | 3.00E-35 | 100.00 | [ATW61781.1](https://www.ncbi.nlm.nih.gov/protein/ATW61781.1?report=genbank&log$=prottop&blast_rank=1&RID=VDBXCEX0014) |
| 67 | 216 | T | Holin | 98 | 4.00E-17 | 98.59 | [AQN32404.1](https://www.ncbi.nlm.nih.gov/protein/AQN32404.1?report=genbank&log$=prottop&blast_rank=3&RID=VDC2WMBM014) |
| 68 | 486 | T | Endolysin | 84 | 6.00E-82 | 100.00 | [YP_006987885.1](https://www.ncbi.nlm.nih.gov/protein/YP_006987885.1?report=genbank&log$=prottop&blast_rank=1&RID=VDC5GPDV014) |
| 69 | 390 | T | Unimolecular spanin | 99 | 4.00E-80 | 93.02 | [QDJ97954.1](https://www.ncbi.nlm.nih.gov/protein/QDJ97954.1?report=genbank&log$=prottop&blast_rank=1&RID=C7MFBZK7016) |
| 70 | 336 | B | Hypothetical protein | 99 | 3.00E-69 | 90.99 | [AQN32388.1](https://www.ncbi.nlm.nih.gov/protein/AQN32388.1?report=genbank&log$=prottop&blast_rank=2&RID=VDD941UG014) |
| 71 | 516 | B | Hypothetical protein | 99 | 7.00E-81 | 66.67 | [YP_008059739.1](https://www.ncbi.nlm.nih.gov/protein/YP_008059739.1?report=genbank&log$=prottop&blast_rank=1&RID=A6JC05KU016) |
| 72 | 1,584 | B | Helicase | 99 | 0 | 83.80 | [YP_009284698.1](https://www.ncbi.nlm.nih.gov/protein/YP_009284698.1?report=genbank&log$=prottop&blast_rank=21&RID=VDDWRHN0015) |
| 73 | 117 | B | Hypothetical protein |  |  |  |  |
| 74 | 240 | B | Hypothetical protein | 98 | 4.00E-43 | 87.34 | [YP_399012.1](https://www.ncbi.nlm.nih.gov/protein/YP_399012.1?report=genbank&log$=prottop&blast_rank=2&RID=VDE9P2WN015) |
| 75 | 354 | B | Hypothetical protein | 98 | 4.00E-65 | 84.48 | [ATW61787.1](https://www.ncbi.nlm.nih.gov/protein/ATW61787.1?report=genbank&log$=prottop&blast_rank=2&RID=VDECM4JH014) |
| 76 | 192 | B | Hypothetical protein | 89 | 2.00E-21 | 78.95 | [AQN32410.1](https://www.ncbi.nlm.nih.gov/protein/AQN32410.1?report=genbank&log$=prottop&blast_rank=1&RID=VDEFTDAA014) |
| 77 | 162 | B | Hypothetical protein | 98 | 3.00E-31 | 98.11 | [QAU04394.1](https://www.ncbi.nlm.nih.gov/protein/QAU04394.1?report=genbank&log$=prottop&blast_rank=1&RID=VDEREXD9014) |
| 78 | 240 | B | Hypothetical protein | 98 | 9.00E-49 | 94.94 | [ATW61790.1](https://www.ncbi.nlm.nih.gov/protein/ATW61790.1?report=genbank&log$=prottop&blast_rank=2&RID=VDFE8U1Y014) |
| 79 | 201 | B | Hypothetical protein | 83 | 8.00E-06 | 35.59 | [SMH63949.1](https://www.ncbi.nlm.nih.gov/protein/SMH63949.1?report=genbank&log$=prottop&blast_rank=1&RID=VDFHNK68015) |
| 80 | 177 | B | Hypothetical protein | 98 | 3.00E-32 | 93.10 | [ATW61791.1](https://www.ncbi.nlm.nih.gov/protein/ATW61791.1?report=genbank&log$=prottop&blast_rank=1&RID=VDFPTMTU014) |
| 81 | 78 | T | tRNA^Arg^ |  |  |  |  |
