# Supplementary material for: Infection Kinetics and Phylogenetic Analysis of vB_EcoD_SU57, a Virulent T1-Like Drexlerviridae Coliphage
Source: Front Microbiol. 2020 Nov 16;11:565556. doi: 10.3389/fmicb.2020.565556 (PMC7718038; doi:10.3389/fmicb.2020.565556)
Supplement: Supplementary Table 2 — T1-like Drexlerviridae (Siphoviridae) phages used for phylogenetic analyses. [file Table_2.DOCX]

Supplementary Table 2. T1-like *Drexlerviridae* (*Siphoviridae*) phages used for phylogenetic analyses. * represents phages in which there are no corresponding published reference articles.

| NCBI Accession Number | Phage | Host  Bacteria | Genome Size (bp) | References |
| --- | --- | --- | --- | --- |
| NC_024784.1 | Escherichia_phage_bV_EcoS_AHS24_complete_genome | *E. coli* | 46,440 | (Niu et al., 2014) |
| NC_019718.1 | Enterobacteria_phage_vB_EcoS_Rogue1_complete genome | *E. coli* | 45,805 | (Kropinski et al., 2012) |
| KC579452.1 | Enterobacteriophage_phiKP26_complete_genome | *E. coli, Salmonella* | 47,285 | (Amarillas et al., 2013b) |
| NC_042046.1 | Escherichia_phage_C119_complete_genome | *E. coli* | 47,319 | (Amarillas et al., 2016) |
| NC_024210.1 | Escherichia_phage_e4/1c_complete_genome | *E. coli* | 47,112 | (O’Flynn et al., 2004) |
| MF630922.1 | Escherichia_phage_IMM-001_complete_genome | *E. coli* | 32,468 | (Chakraborty et al., 2018) |
| NC_007603.1 | Enterobacteria_phage_RTP_complete_genome | *E. coli* | 46,219 | (Wietzorrek et al., 2006) |
| KX130960.1 | Escherichia_phage_vB_EcoS-IME253_complete_genome | *E. coli* | 46,717 | (Li et al., 2019) |
| MG050172.1 | Escherichia_phage_DTL_complete_genome | *E. coli* | 45,814 | (Halter and Zahn, 2018) |
| NC_019404.1 | Enterobacteria_phage_vB_EcoS_ACG-M12_complete_genome | *E. coli* | 46,054 | (Chibeu et al., 2012) |
| KY398841.1 | Escherichia_phage_vB_Ecos_CEB_EC3a_complete_genome | *E. coli* | 44,234 | (Oliveira et al., 2018) |
| NC_005833.1 | Enterobacteria_phage_T1_complete_genome | *E. coli, Shigella* | 48,836 | (Roberts et al., 2004) |
| NC_009540.1 | Enterobacteria_phage_TLS_complete_genome | *E. coli, Shigella* | 49,902 | (German and Misra, 2001) |
| KX130668 | Enterobacteria phage vB_EcoS_NBD2, complete genome | *E.coli* | 51,802 | (Kaliniene et al., 2018) |
| KF771236.1 | Escherichia phage bV_EcoS_AHP24, complete genome | *E. coli* | 46,719 | (Niu et al., 2014) |
| JF770475.1 | Escherichia_phage_phiEB49_complete_genome | *E. coli* | 47,180 | (Battaglioli et al., 2011) |
| MK373793.1 | Escherichia phage vB_EcoS_MM01, complete genome | *E. coli* | 43,157 | (Korf et al., 2019) |
| MF158039.1 | Shigella_phage_Sf12_complete_genome | *Shigella* | 47,647 | (Doore et al., 2018) |
| MF158042.1 | Shigella_phage_Sd1_complete_genome | *Shigella* | 48,262 | (Doore et al., 2018) |
| MK907226.1 | Escherichia phage vB_EcoS-12210I, complete genome | *E. coli* | 44,219 | (Pacífico et al., 2019) |
| NC_024789.1 | Escherichia phage bV_EcoS_AKS96, complete genome | *E. coli* | 45,746 | (Niu et al., 2014) |
| GQ495225.1 | Escherichia phage RES-2009a, partial genome | *E. coli* | 7,513 | (Hobley et al., 2020) |
| NC_019509.1 | Cronobacter_phage_ESP2949-1_complete_genome | *Cronobacter* | 49,116 | (Lee et al., 2012) |
| NC_043469.1 | Enterobacteria_phage_F20_partial_genome | *Enterobacter* | 51,543 | (Mishra et al., 2012) |
| KC333879.1 | Enterobacteria_phage_phiJLA23_complete_genome | *E. coli* | 43,017 | (Amarillas et al., 2013a) |
| [MH845412.1](https://www.ncbi.nlm.nih.gov/nuccore/MH845412.1) | Cronobacter phage CS01 | *Cronobacter sakazakii* | 48,195 | (Kim et al., 2019) |
| NC_024793.1 | Escherichia phage bV_EcoS_AHP42, complete genome | *E. coli* | 46,847 | (Niu et al., 2014) |
| MN164484.1 | Escherichia virus ECH1, complete genome | *E. coli* | 49,553 | * |
| NC_007291.1 | Enterobacteria_phage_JK06_complete_genome | *E. coli* | 46,072 | * |
| MK372342.1 | Enterobacteria phage vB_EcoS_IME542, complete genome | *Enterobacter* | 46,553 | * |
| MN840485.1 | Escherichia phage 2725-N35, complete genome | *E. coli* | 45,917 | * |
| KY619305 | Escherichia phage vB_EcoS_ESCO41, complete genome | *E. coli* | 50,800 | * |
| [NC_042122.1](https://www.ncbi.nlm.nih.gov/nuccore/NC_042122.1) | Enterobacter phage Ec_L1 | *Enterobacter cloacae* | 51,894 | * |
|  |  |  |  |  |
